# Supplementary material for: Electronically driven collapse of the bulk modulus in δ-plutonium
Source: Proc Natl Acad Sci U S A. 2020 Feb 18;117(9):4480–5. doi: 10.1073/pnas.1918281117 (PMC7060724; doi:10.1073/pnas.1918281117)
Supplement: Supplementary File [file pnas.1918281117.sapp.pdf]

PNAS
www.pnas.org

Supplementary Information for

Electronically driven collapse of the bulk modulus in δ-plutonium

Neil Harrison

Neil Harrison
Email: nharrison@lanl.gov

This PDF file includes:

Supplementary text
Figures S1 to S2

www.pnas.org/cgi/doi/10.1073/pnas.1918281117

Supporting Information Text

Prior models of the bulk modulus softening. In a prior model of the bulk modulus based on the invar model, the Debye temperature, and consequently the bulk modulus, was assumed to be the probability-weighted sum of the ground state bulk modulus and an excited invar configuration bulk modulus (1-3), which is equivalent to the first term of Eq. 4 of the main text. In the absence of any other terms contributing to the bulk modulus, Lawson *et al* were able to approximately account for the experimentally observed softening of the bulk modulus by setting $K_i = 0$ for the excited invar configuration. Such a value is expected to occur only at a very large positive volume strain of $\nu = \frac{61}{64} + \nu_i$, which we obtain by equating the second derivative of Eq. 6 in the Methods to zero. A volume strain this large $\nu \sim$ 100% corresponds to an actual volume of order $V \sim$ 50 Å, which is significantly larger than the value $V_i \approx$ 20 Å for the excited configuration of δ-Pu obtained in fitting the invar model to the thermal expansion (1,2).

In another more recent model of the bulk modulus based on the disordered local moment model (4), the volume was assumed to remain approximately constant with the different configurations corresponding to a continuum of states with different degrees of orbital compensation of the local moment (5). In this model, the softening of the bulk modulus occurs in response to a reduction in the moment with increasing temperature. However, since the degree of softening in this model is predicted to be reduced for samples with a larger negative contribution to the thermal expansion, which generally occurs for samples with lower concentrations *x* of Ga (1), the predicted Ga-dependent trend is opposite to that found experimentally (plotted in Fig. 1*B* of the main text) (6).

Applicability of electronic structure methods. Advanced electronic structure methods utilizing Quantum Monte Carlo (QMC) or Dynamical Mean Field Theory (DMFT) inform us of how the 5f-electron shells intermix with conduction electrons (7,8). These methods are essential for an accurate interpretation of spectroscopic measurements such as photoemission and quantum oscillations. However, they do not provide the most practical means for understanding thermodynamic quantities such as the bulk modulus.

A central point of this manuscript is that in order to understand the origin of the softening, the bulk modulus and its pressure derivative must be calculated from the second and third order derivatives, respectively, of the free energy with respect to volume — and over a broad range of temperatures. While QMC and DMFT can be used to calculate the energy-versus-volume curves at different temperatures, to do this energy over a sufficiently dense grid of volumes and temperatures to accurately determine the second and third order volume derivatives would constitute a formidable computational task. A single DMFT calculation performed at a single volume and temperature typically takes of order a week using state-of-the-art code and computer systems. Very approximate treatments have, instead, generally been advocated, which include the invar model (3) and atomistic model (9).

Questions regarding pressure-dependent data. There are uncertainties in how some of the experimental compositions in Fig. 4 of the main text can be compared with the model. For instance, the *x* = 3.5% Ga-stabilized sample, for which $K' \sim$ + 4 is positive in contrast to the other δ-Pu samples (10), has a substantially lower volume ($V \sim$ 24.2 Å) than that ($V \sim$ 24.6 Å) previously found for samples of nominally the same composition (interpolating between *x* = 2 and 4%) (1). Also, the lowest error bar ($K' =$ - 4 ± 2) is obtained for a heavily Am-stabilized δ-Pu sample (likely due to the wider range of accessible pressures), yet its thermal expansion and elastic properties remain largely unexplored (11).

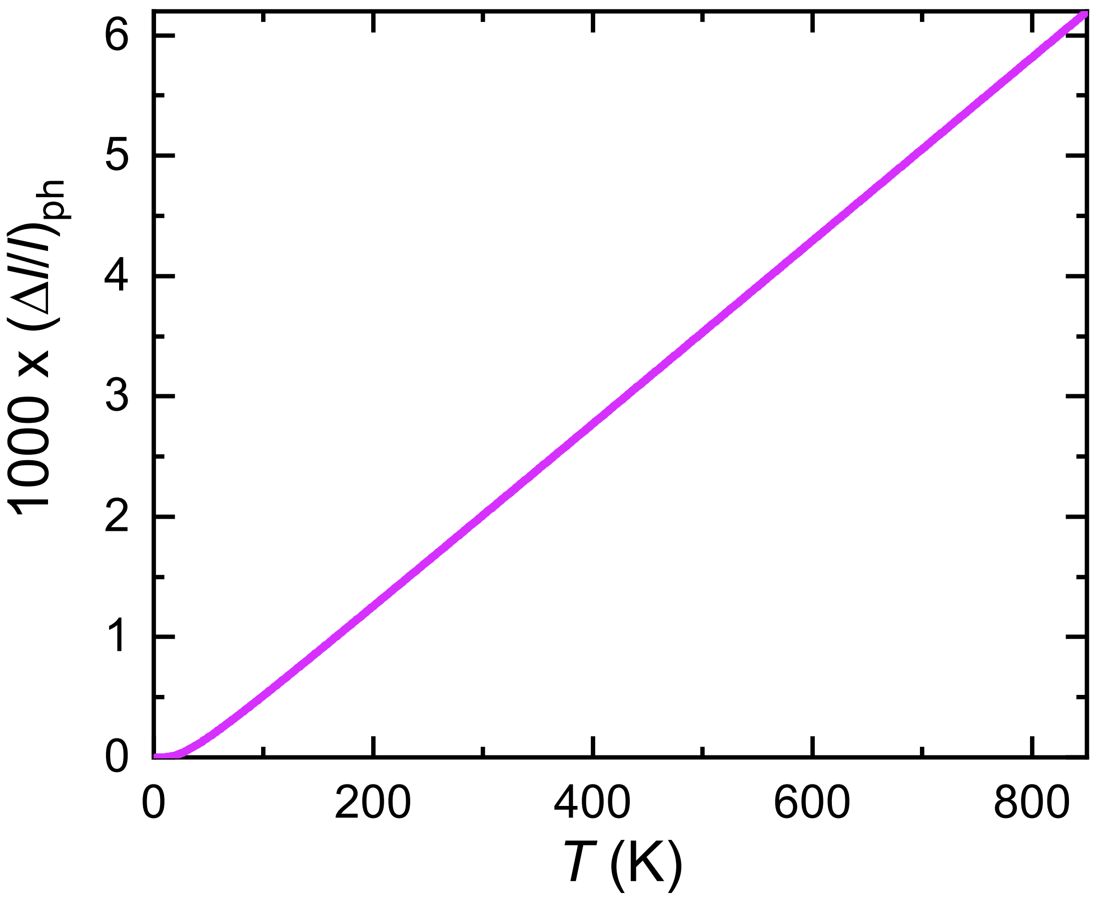

Fig. S1. The linear thermal expansion of δ-Pu attributable to phonons, determined from a combined fit to thermal expansion and magnetostriction data, and its verification using heat capacity measurements (3).

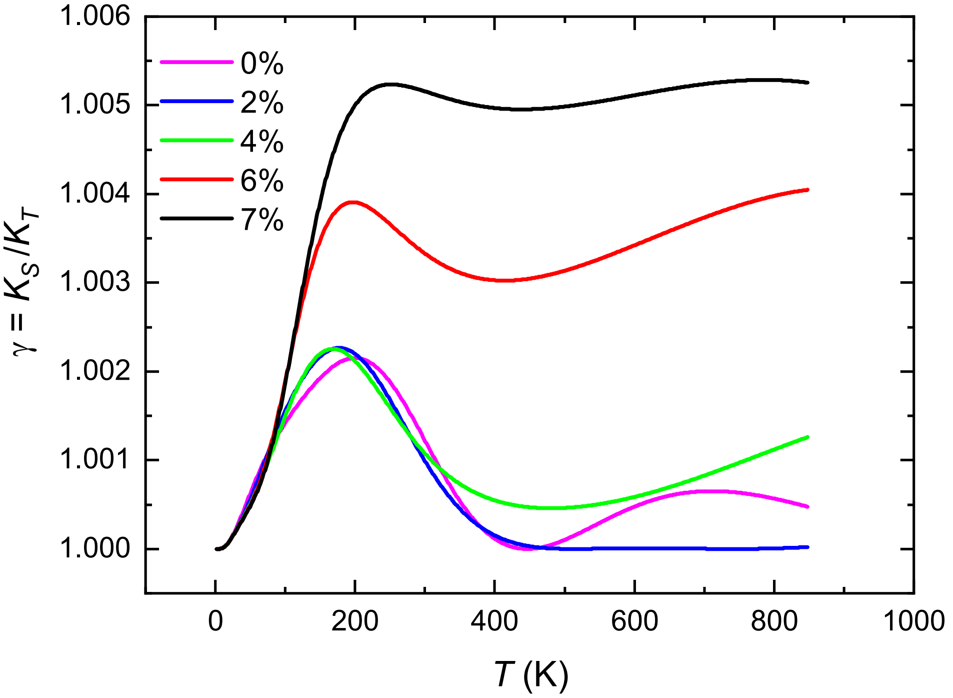

Fig. S2. Calculated ratio $\gamma = \frac{\kappa_S}{\kappa_T} = \frac{C_p}{C_T}$, according to multiple electronic configurations (12), with the concentrations of Ga substituted into δ-Pu indicated in different colors.

SI References

1. A. C. Lawson , J. A. Roberts , B. Martinez, J. W. Richardson, Invar effect in Pu-Ga alloys. *Phil. Mag. B* **82** 1837-1845 (2002).
2. A. C. Lawson, J. A. Roberts, B. Martinez, M. Ramos, G. Kotliar, F. W. Trouw, M. R. Fitzsimmons, M. P. Hehlen, J. C. Lashley, H. Ledbetter, R. J. Mcqueeney, A. Migliori, Invar model for δ-phase Pu: thermal expansion, elastic and magnetic properties. *Phil. Mag*. **86**, 2713-2733 (2006).
3. A. C. Lawson, Thermodynamics of the bulk modulus of delta phase plutonium alloys. *Phil. Mag*. **99**, 1481-1498 (2019).
4. A. Migliori, P. Soderlind, A. Landa, F. J. Freibert, B. Maiorov, B. J. Ramshaw, J. B. Betts, Origin of the multiple configurations that drive the response of δ-plutonium's elastic moduli to temperature. *Proc. Nat. Acad. Sci. USA* **113**, 11158-11161 (2016).
5. P. Söderlind, Quantifying the importance of orbital over spin correlations in δ-Pu within density-functional theory. *Phys. Rev. B* **77**, 085101 (2008).
6. P. Söderlind, A. Landa, J. E. Klepeis, Y. Suzuki, A. Migliori, Elastic properties of Pu metal and Pu-Ga alloys. *Phys. Rev. B* **82**, 224110 (2010).
7. J.-X. Zhu, A. K. McMahan, M. D. Jones, T. Durakiewicz, J. J. Joyce, J. M. Wills, R. C. Albers, Spectral properties of δ-plutonium: Sensitivity to 5*f* occupancy, *Phys. Rev. B* **76**, 245118 (2007).
8. J. H. Shim, K. Haule, G. Kotliar, Fluctuating valence in a correlated solid and the anomalous properties of δ-plutonium. *Nature* **446**, 513-516 (2007).
9. Z. P. Yin, X. Deng, K. Basu, Q. Yin, G. Kotliar, Temperature-dependent electronic structures, atomistic modelling and the negative thermal expansion of δ Pu. *Phil. Mag. Lett*. **94**, 620-628 (2014).
10. J. Zhang, F. J. Freibert, B. Clausen, A. I. Smith, S. C. Vogel, D. W. Brown, Equation of state and strain-induced stabilization δ-phase stabilized plutonium alloys. *J. Nucl. Mater.* **524**, 54 (2019).
11. S. S. Hecker, D. R. Harbur, T. G. Zocco, Phase stability and phase transformations in Pu-Ga alloys. *Prog. Mater. Science* **49**, 429-485 (2004).
12. N. Harrison, J.B. Betts, M.R. Wartenbe, F.F. Balakirev, S. Richmond , M. Jaime, P.H. Tobash, Phase stabilization by electronic entropy in plutonium. *Nature Commun*. **10**, 3159 (2019).
